# Sex differences in trajectories of cortical development in autistic children from 2–13 years of age

**DOI:** 10.1038/s41380-024-02592-8

**Published:** 2024-05-16

**Authors:** Derek S. Andrews, Kersten Diers, Joshua K. Lee, Danielle J. Harvey, Brianna Heath, Devani Cordero, Sally J. Rogers, Martin Reuter, Marjorie Solomon, David G. Amaral, Christine Wu Nordahl

**Affiliations:** 1 Department of Psychiatry & Behavioral Sciences, the MIND Institute, University of California, Davis, CA USA; 2 AI in Medical Imaging, German Center for Neurodegenerative Diseases, Bonn, Germany; 3 Division of Biostatistics, Department of Public Health Sciences, University of California, University of California, Davis, CA USA; 4 A.A. Martinos Center for Biomedical Imaging, Massachusetts General Hospital, Boston, MA USA; 5 Department of Radiology, Harvard Medical School, Boston, MA USA

**Keywords:** Autism spectrum disorders, Neuroscience

## Abstract

Previous studies have reported alterations in cortical thickness in autism. However, few have included enough autistic females to determine if there are sex specific differences in cortical structure in autism. This longitudinal study aimed to investigate autistic sex differences in cortical thickness and trajectory of cortical thinning across childhood. Participants included 290 autistic (88 females) and 139 nonautistic (60 females) individuals assessed at up to 4 timepoints spanning ~2–13 years of age (918 total MRI timepoints). Estimates of cortical thickness in early and late childhood as well as the trajectory of cortical thinning were modeled using spatiotemporal linear mixed effects models of age-by-sex-by-diagnosis. Additionally, the spatial correspondence between cortical maps of sex-by-diagnosis differences and neurotypical sex differences were evaluated. Relative to their nonautistic peers, autistic females had more extensive cortical differences than autistic males. These differences involved multiple functional networks, and were mainly characterized by thicker cortex at ~3 years of age and faster cortical thinning in autistic females. Cortical regions in which autistic alterations were different between the sexes significantly overlapped with regions that differed by sex in neurotypical development. Autistic females and males demonstrated some shared differences in cortical thickness and rate of cortical thinning across childhood relative to their nonautistic peers, however these areas were relatively small compared to the widespread differences observed across the sexes. These results support evidence of sex-specific neurobiology in autism and suggest that processes that regulate sex differentiation in the neurotypical brain contribute to sex differences in the etiology of autism.

## Introduction

Autism spectrum disorder (ASD or autism) is a neurodevelopmental condition characterized by challenges with social interaction and communication, and repetitive restricted behaviors [1]. Approximately four males are diagnosed with autism for every one female [2], which has led autistic females to be historically underrepresented in autism research, including neuroimaging studies [3]. Increased research focus on autistic females has resulted in the identification of several neural sex differences in autism [4–21]. However, neuroimaging studies starting from the earliest age of autism diagnosis are exceedingly rare and the extent of sex differences in autistic neural development across childhood is unknown.

Several autism neuroimaging studies have focused on the structure of the cerebral cortex, particularly its thickness. In typical development, mean cortical thickness increases rapidly across the prenatal period, peaks between 1.5–2 years of age, and is followed by a prolonged period of cortical thinning across the lifespan [22–24]. While this is generally true, it has become clear that patterns of cortical thickness differences in autism have high degrees of interindividual variability [25, 26]. Large, cross-sectional MRI studies have reported both increased and decreased cortical thickness in autism across multiple cortical regions. These studies, while cross-sectional in nature, have suggested that differences in cortical thickness in autism are somewhat age dependent and can vary across developmental stages. For example, autistic individuals have been reported to have regional variability in the age at which peak cortical thickness is achieved, slower rates of cortical thinning with age, and larger differences in cortical thickness from neurotypical individuals at younger ages and during adolescence compared to later in life [6, 25, 27, 28]. Longitudinal studies of the trajectory of cortical thinning in autistic children are limited and have included all male, or predominately male, participants. One such study suggests that, compared to nonautistic males, autistic males experience accelerated thickening prior to 3 years of age followed by accelerated thinning through the remainder of childhood [29]. However, another longitudinal study of mostly males reported less cortical thinning from 4–6 years of age in autistic individuals [30]. Longitudinal assessment of cortical development in autistic individuals with an adequate sample of female participants may resolve some of the remaining gaps in our understanding of sex specific differences in autism. For example, we previously showed that cerebral overgrowth in autism affected a larger proportion of males and was persistent across development, while autistic females had slower grey and white matter growth trajectories throughout early childhood [14, 31, 32].

Several theories have been proposed to explain differences in autism prevalence between the sexes. Prominent among these are the multifactorial liability model and “female protective effect” [33]. These propose that genetic and environmental risk factors required to develop autism are greater, on average, in females. The concept of the female protective effect has been supported by findings of higher rates of genetic variants associated with autism among autistic females [10, 12, 34, 35]. There is also some evidence of larger brain alterations for autistic females compared to males [5, 7–13, 15, 21]. Accordingly, one could hypothesize these effects extend to cortical structure, with both quantitative (i.e., extent) and/or qualitative (i.e., regions affected) autistic differences between the sexes that occur within regions undergoing sex differentiation in neurotypical development [20]. While some evidence suggests that autistic females have greater increases in, and stronger associations between, cortical thickness and autism symptoms than autistic males, and that different cortical regions are affected between the sexes [6], several recent autism studies have reported no sex differences in cortical thickness [20, 26, 28, 36].

To our knowledge, no study of autism has investigated sex differences in cortical thickness and/or trajectory of cortical thinning longitudinally across childhood. We have evaluated magnetic resonance imaging (MRI) data from the MIND Institute Autism Phenome Project with significant representation of autistic females that spans multiple timepoints from early to late childhood. Based on the literature and our previous research we hypothesized that: (1) autistic compared to nonautistic individuals have significant differences in both cortical thickness and the trajectory of cortical thinning across childhood, (2) the differences associated with autism will be quantitatively greater in autistic females and may be more widespread in autistic females compared to males (3) regions in which autism effects differ between the sexes will overlap with regions that are different between the sexes in neurotypical development, (4) there will also be areas of cortical differences in autism that are shared across males and females, and these regions may be particularly germane to autism.

## Materials and Methods

### Participants

This study included 290 autistic (202 males, 88 females) and 139 nonautistic, typically developing (TD) (79 males, 60 females) individuals (Table 1). Sex assigned at birth was utilized to categorize sex in this study. Participants were enrolled in the ongoing UC Davis MIND Institute Autism Phenome Project (APP) which includes the Girls with Autism Imaging of Neurodevelopment (GAIN) study, launched to increase representation of females within the larger APP. The study design consists of longitudinal MRI scanning at four timepoints: enrollment and baseline at 24–42 months of age (Time One); subsequent follow up at annual intervals for two additional time points (Time Two & Three); and a fourth timepoint between the ages of 8–13 years (Time Four) [37]. Diagnosis of autism spectrum disorder (ASD) was confirmed at study entry using the Autism Diagnostic Observation Schedule-Generic (ADOS-G) [38] or ADOS-2 [39], the Autism Diagnostic Interview-Revised (ADI-R) [40] and DSM-IV-TR criteria [41]. In 12 cases, due to face mask requirements, ASD diagnosis was confirmed using the Brief Observation of Symptoms of Autism (BOSA) [42]. The current study included individuals in the APP cohort who successfully completed a structural MRI scan of sufficient quality to complete the *Freesurfer* preprocessing pipeline [43] for at least one of the four timepoints (Supplementary Methods). Informed consent was obtained from the parent or guardian of each participant. All aspects of the study protocol were approved by the University of California Davis Institutional Review Board.

**Table 1:** Sample Characteristics.

	ASD	TD
*n*	290	139
Male/female	202/88	79/60
Age in months at T1	38.55 (5.91)	37.84 (6.33)
DQ at T1	64.09 (21.41)	106.13 (11.79)
ADOS CSS at T1	7.45 (1.73)	–
ADOS SA at T1	6.91 (1.60)	–
ADOS RRB at T1	8.33 (1.55)	–
Average # timepoints	2.04	2.33
With 1 timepoint	108	37
With 2 timepoints	84	43
With 3 timepoints	74	35
With 4 timepoints	24	24
Total timepoints (male/female)	415/179	183/141
Scans at T1 (male/female)	183/79	72/54
Scans at T2 (male/female)	113/49	47/39
Scans at T3 (male/female)	73/34	34/27
Scans at T4 (male/female)	46/17	30/21

*n* number of participants included after quality assurance, *ASD* autism spectrum disorder, *TD* nonautistic typically developing, *T1-4* Study Times One-Four, *DQ* Mullen Scales of Early Learning Developmental Quotient, *ADOS* Autism Diagnostic Observation Schedule, *CSS* calibrated severity score, *SA* social affect, *RRB* restricted repetitive behaviors.

### Image acquisition and processing

All MRI scanning was performed at the UC Davis Imaging Research Center, using a 3 Tesla Siemens Magnetom Trio MR system (Erlangen, Germany) with an 8-channel head coil. High resolution T1 images were acquired for Time One - Three scans during natural nocturnal sleep without sedation [44]. Time Four scans were acquired when participants were awake utilizing principles of applied behavior analysis to improve compliance [45], in addition to a modified sequence to shorten scanning duration.

Cortical thickness was estimated from cortical surface reconstructions of structural MRI scans using Freesurfer v7.1.1 [43, 46]. These methods have been extensively described elsewhere [47, 48], and results validated histologically [49]. All surface reconstructions were visually inspected for quality and, when appropriate, manual edits were performed to improve reconstruction quality. Of 1115 eligible MRI timepoints a total of 197 (15%) were excluded due to quality issues that could not be corrected by manual edits and are not described in this study (Supplementary Methods).

### Statistical analysis

We first evaluated differences in the trajectory of cortical thinning between autistic females and nonautistic females compared to differences between autistic males and nonautistic males. This contrast (i.e., age-by-sex-by-diagnosis interaction) highlights differences in *trajectory of cortical thinning* associated with autism *between the sexes*. Models were then re-estimated with age centered at the mean ages of Time One (38.26 months/3.19 years), Time Three (63.98 months/5.33 years), and Time Four (137.31 months/11.44 years) allowing the investigation of differences in *cortical thickness* associated with autism between the sexes at these specific ages (i.e., sex-by-diagnosis interaction). *Within sex* contrasts for cortical thickness at Time One, Time Three, Time Four, and for trajectories of cortical thinning between autistic and nonautistic individuals were then leveraged to aid in the interpretation of sex differences and identify cortical regions where effects associated with autism were shared across the sexes.

Vertex-wise longitudinal modeling of cortical thickness development was performed using spatiotemporal linear mixed-effects models controlling for multiple comparisons using a two-stage adaptive false discovery rate (FDR) procedure within the *fslmer* package in R v4.2.1 [50–52]. Models included a three-way interaction of age-by-sex-by-diagnosis and all lower order two-way interactions and main effects, with individual random intercepts and random slopes with age. Age was modeled by selecting from a range of single order polynomial terms (−3, −2, −1, −0.5, 0.5, 1, 2, 3) that returned the lowest log likelihood when modeling whole brain mean cortical thickness [53]. 1/age was determined to provide the best fit and was thus utilized for all subsequent models. Given our group’s prior investigations of autistic subgroups based on brain volumes [14, 31, 32], as well as the current inclusion of a representative number of autistic individuals with DQ in the range of intellectual disability, analyses were repeated using models that also included total cortical volume and developmental quotient (DQ) at age of enrollment as covariates [54]. We additionally repeated analyses when including a quantitative measure of the number of topological defects (i.e., *SurfaceHoles*) in each Freesurfer cortical reconstruction as a covariate [47]. Models with and without these covariates provided generally comparable results (Supplementary Figs. 1, 2).

Significant effects were interpreted within the context of seven functional neural networks defined by Yeo et al. [55] (i.e., visual, somatomotor, dorsal attention, ventral attention, limbic, frontoparietal, and default mode). For each contrast of interest, we report the total area of significant differences in cortical thickness or trajectories of cortical thinning in terms of the entire cortical surface (i.e., percent of the cortical surface affected) and across these functional networks (i.e., percent of the functional network affected). Specific brain regions were identified according to the Desikan cortical atlas [56]. All brain maps were visualized using *fsbrain* in R [57].

The similarity between cortical statistical maps of neurotypical sex differences and sex-by-diagnosis differences were directly tested using spin tests implemented in MATLAB version 9.12.0 2022a [58]. In brief, the spin test evaluates spatial correspondence between brain maps by generating a null model of overlap using a spatial permutation framework in which cortical maps are permutated through random rotations in a spherical space [59]. Here four map pairs were compared (*n* = 1000 permutations of *F* maps); 1. age-by-sex-by-diagnosis v TD age-by-sex, 2. Time One sex-by-diagnosis v Time One TD sex, 3. Time Three sex-by-diagnosis v Time Three TD sex, and 4. Time Four sex-by-diagnosis v Time Four TD sex.

## Results

### Participant characteristics

Autistic and nonautistic children did not significantly differ in age at any of the four time points (*p* > 0.05). The autistic sample contained a higher proportion of male individuals (2.29:1) compared to the nonautistic sample (1.31:1) (χ^2^ = 6.27, *p* = 0.01). At enrollment (Time One), autistic children had significantly lower Mullen DQ scores compared to nonautistic children (*p* < 0.001). At Time One, autistic males and females did not differ significantly in full scale DQ scores nor ADOS total, social affect or restricted repetitive behavior calibrated severity scores (*p* > 0.05). On average, autistic children completed 2.04 total scanning timepoints, which was significantly less than the 2.33 completed by nonautistic children (*p* = 0.008) (Table 1).

### Autistic sex differences in the trajectory of cortical thinning

**Fig. 1:**
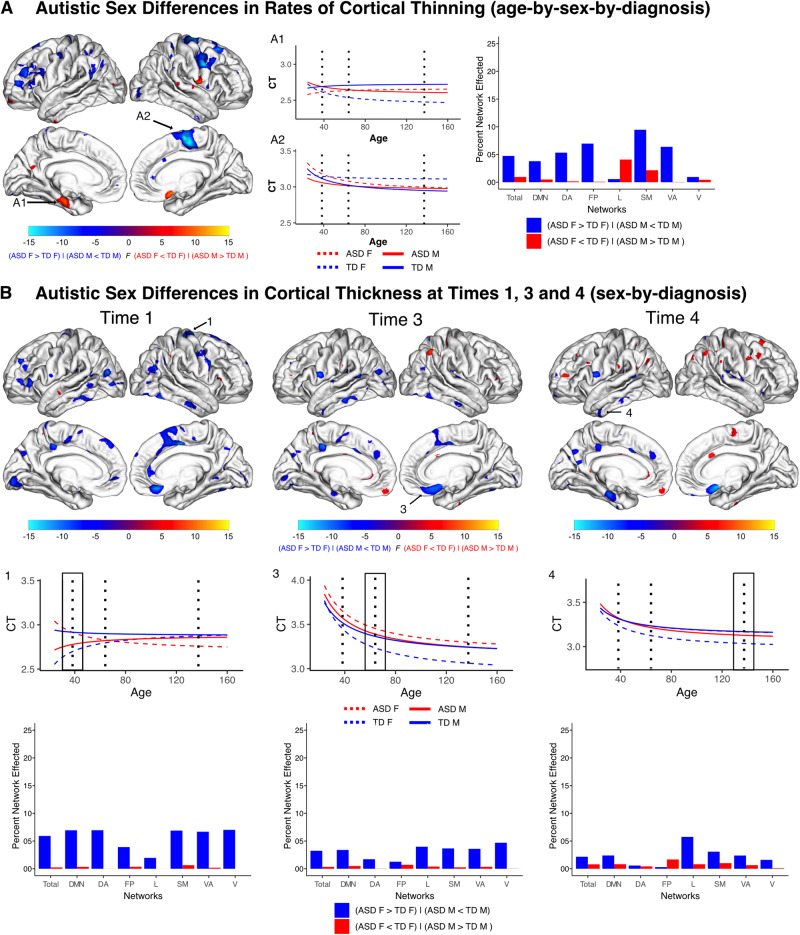
Autistic sex differences in rates of cortical thinning and cortical thickness across childhood. Cortical regions with significant sex-by-diagnosis interactions in trajectories of cortical thinning (**A**) and in cortical thickness at Times One, Three and Four (**B**). Line plots indicate trajectories for highlighted clusters, vertical dotted lines indicate mean ages at Times One, Three and Four. Bar plots indicate proportion of functional networks affected (total total cortical surface, DMN default mode, DA dorsal attention, FP frontal parietal, L imbic, SM somatomotor, VA ventral attention, V visual). **A** Significant age-by-sex-by-diagnosis interactions in cortical thinning trajectories were predominately identified as regions with faster thinning in autistic (ASD) females and slower thinning in ASD males compared to sex matched typically developing nonautistic (TD) individuals. These clusters involved 4.74% of the total cortex and were distributed across all seven functional networks, but most proportionately affected the SM (9.46%), FP (6.96%), VA (6.38%), and DA (5.33%). In contrast, regions where autistic females had slower and autistic males had faster rates of cortical thinning incorporated 0.95% of the total cortical surface, most proportionately affecting the L (4.08%) and SM (2.15%). **B** Significant sex-by-diagnosis interactions in cortical thickness at Time One were predominately characterized by regions of thicker cortex in autistic females and thinner cortex in autistic males compared to sex matched TD individuals. These clusters incorporated 5.92% of the total cortical surface, most proportionately affecting the V (7.02%), DA (6.95%), DMN (6.94%), SM (6.88%), and VA (6.68%). Regions where autistic females had thinner cortex and autistic males thicker cortex were isolated to select regions comprising 0.25% of the total cortical surface. Across childhood faster cortical thinning across several cortical regions for autistic females resulted in shifts in the spatial extent of significant sex-by-diagnosis interactions at Time Three and Four. By Time Four, regions in which autistic females had thicker cortex and autistic males thinner cortex reduced to 2.17% of the total cortical surface, most proportionately affecting the L (5.75%), SM (3.08%), DMN (2.26%) and VA (2.38%). Additionally, at Time Four regions in which autistic females had thinner cortex and autistic males thicker cortex increased to 0.80% of the total cortical surface and most proportionately affected the FP (1.67%) and SM (1.01%).

Results of the three-way interaction between age, sex, and diagnosis revealed several cortical regions with opposing trajectories of cortical thinning associated with autism between males and females (Fig. 1A). Autistic compared to nonautistic females primarily had regions of more rapid cortical thinning across childhood while autistic males had less rapid thinning compared to nonautistic males (ASD F > TD F & ASD M < TD M). These findings were distributed across several brain regions and neural networks. In aggregate, this involved 4.74% of the cortical surface, with the somatomotor, frontoparietal, and ventral attention networks being the most affected proportionally. Significant differences in the opposite direction, whereby autistic males had accelerated thinning compared to nonautistic males and autistic females had less rapid thinning compared to nonautistic females (ASD M > TD M & ASD F < TD F), occurred in 0.95% of the cortical surface with the limbic network being the most proportionally affected.

**Fig. 2:**
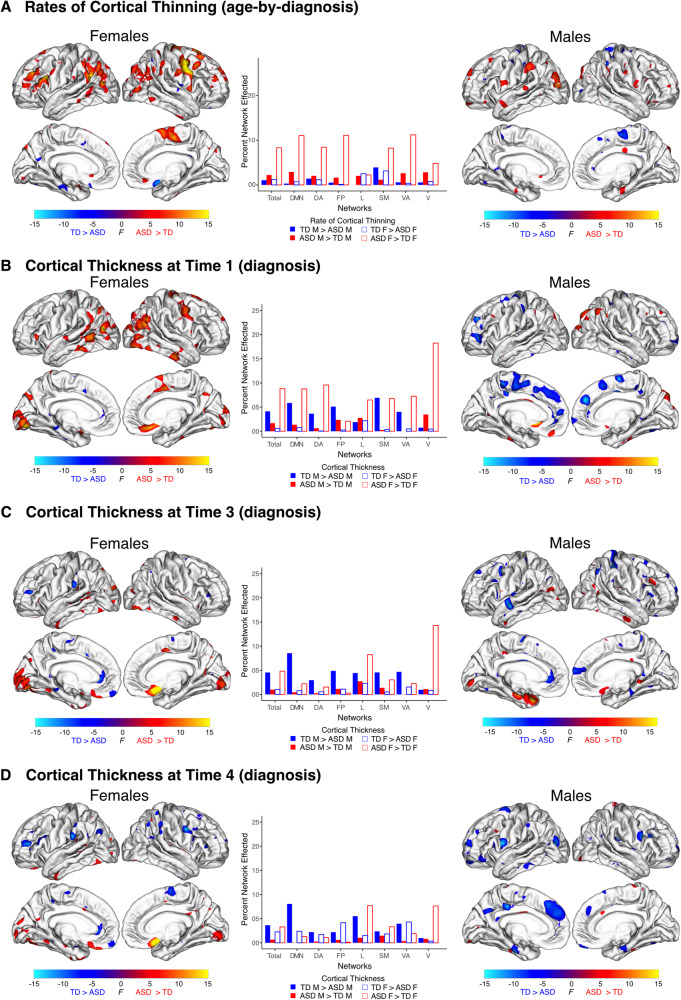
Within sex effects of autism on rates of cortical thinning and cortical thickness across childhood. Cortical regions with significant effects of autism diagnosis between autistic and nonautistic females, and between autistic and nonautistic males in rates of cortical thinning (**A**) and in cortical thickness at Times One (**B**), Three (**C**), and Four (**D**) are highlighted. Bar plots indicate proportion of functional networks affected within each sex (total total cortical surface, DMN default mode, DA dorsal attention, FP frontal parietal, L limbic, SM somatomotor, VA ventral attention, V visual). **A** Autistic females had significantly faster rates of cortical thinning from Time One - Four in several regions compared to autistic males (8.32% of cortex in females compared to 2.21% in males), in contrast autistic males and females had a comparable number of regions with less thinning than nonautistic participants of the same sex (1.05% of cortex in males compared to 1.18% in females). **B** At Time One autistic females predominately had significant increases in cortical thickness across several cortical regions. This was in contrast to autistic males whom at Time One had less regions of thicker cortex (8.83% of cortex in females compared to 1.67% in males) and more regions with thinner cortex (4.12% of cortex in males compared to 0.60% in females). **C**, **D** Faster thinning from Time One to Four in autistic females resulted in a reduction in cortical regions with thicker cortex at Times Three (4.81%) and Four (3.29%) compared to Time One, and more regions with thinner cortex (Time Three = 1.01%, Time Four = 2.26%). Autistic males continued to have regions with both significant increases and decreases in cortical thickness in comparative proportions across the cortex at both Times Three (0.99% thicker, 4.54% thinner) and Four (0.66% thicker, 3.65% thinner).

Comparisons within each sex further highlight these differences. Autistic females had more rapid cortical thinning relative to nonautistic females across 8.32% of the cortical surface and less rapid thinning across 1.18% of the cortex. In contrast, within sex differences between autistic and nonautistic males encompassed a smaller proportion of cortex, with significantly more rapid cortical thinning in autistic males in 2.21% and less rapid thinning in 1.05% of the entire cortex (Fig. 2A). Thus, the autistic differences in females that were not observed in males largely contribute to the significant sex differences in the three-way age-by-diagnosis-by-sex interaction described above (Supplementary Tables 1–7).

### Autistic sex differences in cortical thickness in early and late childhood

At Time One (~38 months or 3 years), the predominant findings were characterized by regions in which autistic females had increased cortical thickness compared to nonautistic females, whereas autistic and nonautistic males did not (sex-by-diagnosis interaction at Time One). These regions comprised 5.92% of the cortical surface, with the default mode, dorsal attention, and visual networks most proportionately affected. Regions in which autistic males had increased cortical thickness but females did not were limited to 0.25% of the cortical surface (Fig. 1B).

Follow up within sex comparisons of cortical thickness further highlighted autistic sex differences. At Time One, autistic, compared to nonautistic, females had increased thickness across 8.83% of the cortical surface and decreased cortical thickness across only 0.6% of the cortical surface. In contrast, the most prominent difference between autistic and nonautistic males was regions of decreased cortical thickness (4.12% of the total cortical surface), with regions of thicker cortex encompassing only 1.67% of cortex (Fig. 2B). These sex specific differences show that patterns of thicker cortex in females not seen in males are the primary driver of sex differences (i.e., the sex-by-diagnosis interaction) in cortical thickness at Time One (Supplementary Tables 8–14).

At Time Three (~64 months or 5.3 years), sex specific autistic differences in cortical thickness was reduced in spatial extent from 6.17% at Time One to 3.59%. At this age, diagnosis-by-sex effects continued to be predominantly characterized by regions of increased cortical thickness in autistic compared to nonautistic females not observed between autistic and nonautistic males. A similar trend was noted in sex specific comparisons of cortical thickness at Time Three with autistic females continuing to have more regions of thicker cortex compared to non-autistic females but to a lesser spatial extent than observed at Time One (4.81% compared to 8.83% of the cortex). Differences in cortical thickness between autistic and nonautistic males at Time Three were relatively comparable to Time One findings in terms of direction and spatial extent. (Supplementary Tables 15–21).

At Time Four (~137 months or 11.5 years), autistic individuals continued to have sex specific alterations in cortical thickness (sex-by-diagnosis interaction at Time Four) though these involved only 2.97% of the cortex, a reduction from 6.17% at Time One and 3.59% at Time Three. In a majority of these regions, which most proportionally affected the limbic network, autistic females had thicker cortex than nonautistic females, but autistic and nonautistic males did not. In contrast, the amount of cortex at Time Four which was thinner in autistic females and thicker in autistic males had increased to 0.8% (from 0.25% at Time One) of the cortical surface (most proportionately affecting the frontoparietal network) (Fig. 1C). We attribute these differences at Time Four from Time One to the increased rates of cortical thinning in several of these regions observed in autistic females.

Within sex differences in cortical thickness associated with autism at Time Four further highlight outcomes of sex-specific differences in rates of cortical thinning across childhood. Due to the accelerated trajectory of cortical thinning in autistic females compared to nonautistic females from Time One to Four, by Time Four autistic females had less cortex with increased thickness relative to nonautistic females (i.e., 3.29% at Time Four vs. 8.83% at Time One) and a greater percentage of cortex that was thinner (i.e., 2.26% at Time Four vs. 0.6% at Time One) (Fig. 2C). While at Time Four autistic males continued to have thinner cortex across 3.65% and thicker cortex across only 0.66% of the total cortical surface relative to nonautistic males (Fig. 2B) (Supplementary Tables 22–28).

### Spatial overlap between sex by diagnosis differences and neurotypical sex differences

**Fig. 3:**
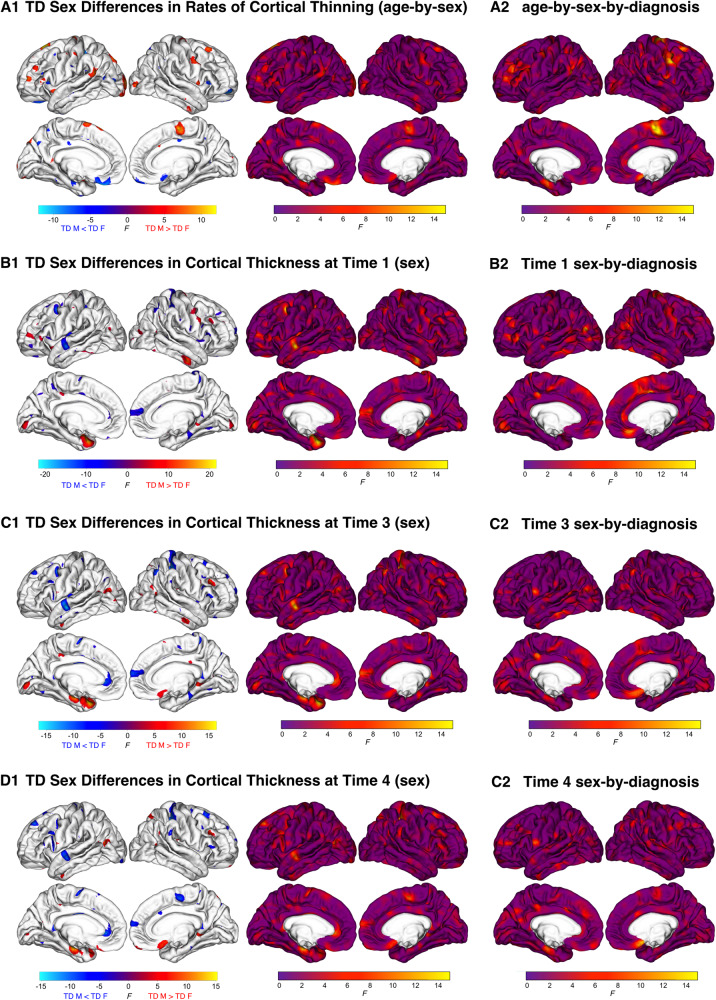
Spatial Correspondence of typical sex differences and sex-by-diagnosis effects in rates of cortical thinning and cortical thickness across childhood. Spatiotemporal FDR corrected and uncorrected *F* maps of typically developing (TD) sex differences in cortical thinning trajectories (A1), and cortical thickness at Times One (B1), Three (C1) and Four (D1). Spin tests were used to test the spatial correspondence of these uncorrected F maps with maps of diagnosis-by-sex interactions in cortical thinning trajectory (A2), and cortical thickness at Times One (B2), Three (C2), and Four (D2). Spin tests (*n* = 1000 permutations) were found to be statistically significant for all three measures (*p* < 0.001), indicating significant overlap between regions which display typical sex differences and sex-by-diagnosis interactions in cortical thinning trajectories and cortical thickness measures across childhood.

Spin tests revealed significant overlap in the spatial extent of the statistical maps depicting sex-specific differences in autistic development (i.e., sex-by-diagnosis-by-age interaction) with cortical regions that have sexually dimorphic developmental differences in neurotypical individuals (i.e., nonautistic age-by-sex) (Fig. 3, Supplementary Tables 29–36). We also observed similarly significant overlap when comparing statistical maps at each time point separately (i.e., Time One, Three, and Four diagnosis-by-sex vs. Time One & Four TD sex) (all *p* < 0.001). This indicates that sex specific autistic differences in cortical thickness and the trajectory of cortical thinning across childhood overlap to a significant degree with cortical regions that exhibit sex differences in nonautistic individuals.

### Similarities between autistic males and females in cortical thickness and development

**Fig. 4:**
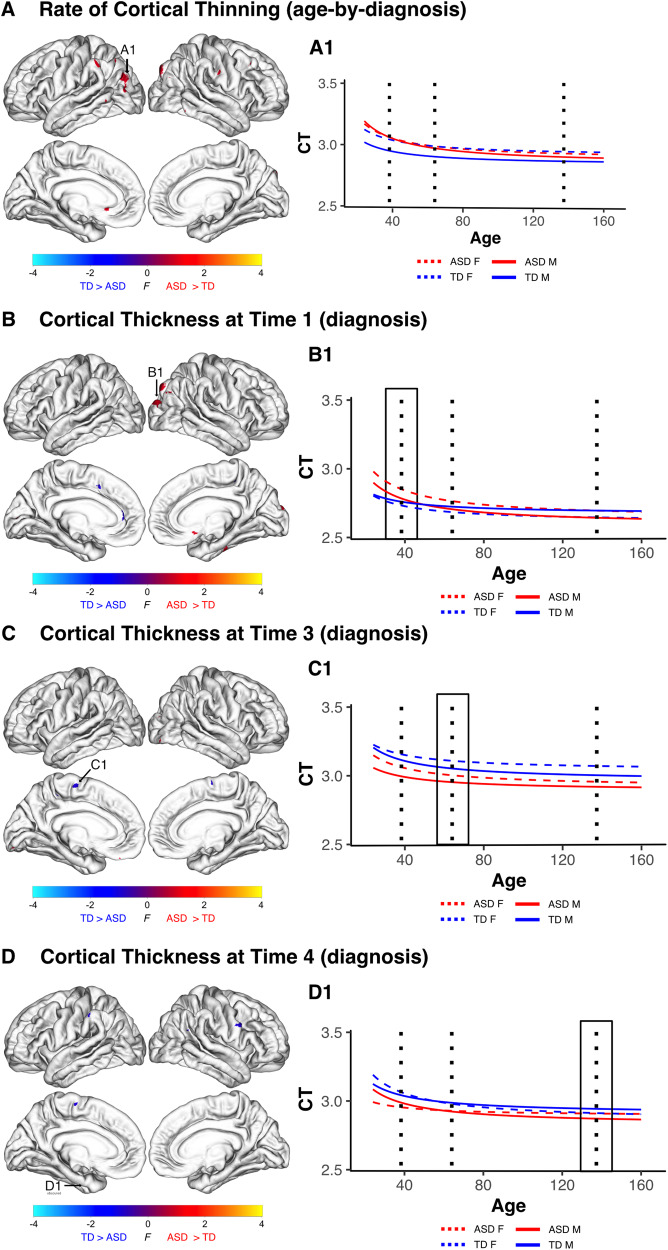
Shared Differences in Cortical Thickness and Thinning Between Autistic Males and Females. **A** Autistic males and females both had clusters of increased thinning centered within regions including left inferior parietal, middle temporal, lateral occipital, regions, the left supramarginal gyrus and rostral anterior cingulate, as well at regions of the right superior parietal, middle frontal, and postcentral gyri. **B** At Time One autistic males and females both had significantly thinner left parahippocampal and superior frontal gyri, as well as increased thickness in right inferior parietal, superior parietal, lateral occipital, medial orbitofrontal, praracentral, and fusiform gyri. **C** At Time Three regions in which autistic males and females both had thinner cortex included bilateral parahippocampal and paracentral gyri as well as the left rostral middle frontal gyrus. Thicker cortex at Time Three was observed in autistic males and females within bilateral lateral occipital cortex and the right fusiform and inferior temporal gyri. **D** At Time Four autistic males and females both had decreased cortical thickness with clusters centered bilaterally in the parahippocampal gyrus, as well as the left supramarginal, paracentral and middle frontal gyrus and right precentral and inferior parietal regions. Additionally, at Time Four both autistic males and females had increased cortical thickness within clusters centered in the right fusiform and superior temporal gyri.

In addition to evaluating sex differences in cortical structure, we also hypothesized that there would be areas of altered cortical development that are common in autistic males and females. In contrast to the widespread differences across males and females described above, the overlap between within-sex female and male maps (i.e., autistic males vs nonautistic males and autistic females vs nonautistic females) were localized to comparatively small focal cortical areas in which autistic males and females had similar significant differences in cortical thinning trajectories or cortical thickness. These areas were characterized by more rapid thinning in autistic individuals of both sexes and were located the left inferior parietal, middle temporal, lateral occipital, supramarginal gyrus, rostral anterior cingulate, as well at regions of the right superior parietal, middle frontal, and postcentral gyri. Collectively these regions in which autistic males and females both had significant age-by-diagnosis effects, in the same direction, comprised just 0.37% of the cortical surface. (Fig. 4A).

At Time One, autistic individuals of both sexes had thicker cortex in clusters located in the right inferior parietal, superior parietal, lateral occipital, medial orbitofrontal, praracentral, and fusiform gyri (0.34% of cortex); areas of thinner cortex were located in left parahippocampal and superior frontal gyri, as well as the left rostral anterior cingulate accounting for 0.14% of the cortical surface (Fig. 4B). At Time Three autistic males and females both had thinner cortex in bilateral parahippocampal and paracentral gyri as well as the left rostral middle frontal gyrus. Thicker cortex at Time Three was observed in autistic males and females within bilateral lateral occipital cortex as well as the right fusiform and inferior temporal gyri (Fig. 4C). At Time Four, autistic males and females both had areas of decreased cortical thickness in bilaterally within the parahippocampal gyrus, as well as the left supramarginal, paracentral and middle frontal gyrus and right precentral and inferior parietal regions accounting for 0.12% of the cortical surface. There were also small clusters of increased cortical thickness in the right fusiform and superior temporal gyri accounting for 0.03% of the cortical surface (Fig. 4D) (Supplementary Tables 37–45).

## Discussion

The overarching goal of this study was to determine whether there are sex-specific differences in cortical development in autistic children. Using longitudinal MRI data from the UC Davis MIND Institute Autism Phenome Project, we evaluated cortical thickness and trajectory of cortical thickness changes from entry into the study at approximately 3 years of age through a fourth study time point at approximately 11.5 years of age. We had several hypotheses concerning the outcomes of this study. Our first was that there would be differences in cortical thickness and trajectory of cortical thinning in autistic individuals compared to nonautistic individuals. This proved to be true more so during early childhood than in late childhood. Our findings are consistent with our second hypothesis, based on the multifactorial liability model, that autistic females would exhibit larger and more extensive cortical differences relative to nonautistic females than autistic males. We observed widespread sex differences in cortical thickness that were most striking at 3 years of age, with autistic females exhibiting increased cortical thickness relative to nonautistic females while differences in autistic males encompassed a smaller spatial extent (and were primarily characterized by thinner cortex). This was followed by sex differences in the trajectory of cortical thinning such that autistic females had widespread regions of accelerated cortical thinning over childhood, whereas autistic males had slower rates of thinning in fewer regions. Interestingly, by middle childhood around 11.5 years of age, sex specific alterations in cortical thickness were less prominent.

The multifactorial liability model suggests that neural sex differences in autism may be characterized by larger effects in females and/or by differences between the sexes in brain regions associated with autism [20]. The current findings highlight a large degree of spatial dissimilarity between the sexes for effects of autism on cortical thickness and rates of cortical thinning. Additionally, within these differentially affected regions females often have quantitatively larger differences (i.e., effect sizes, particularly for differences in rates of cortical thinning). Thus, the current results suggest that sex can modulate autistic neurobiology both qualitatively *and* quantitatively, with affected regions differing spatially between the sexes and categorized by larger effects in autistic females. Furthermore, the increased spatial distribution and magnitude of differences in cortical thickness associated with autism in females may be indicative of a higher liability threshold for females to develop autism, and thus lends support to multifactorial liability models of autism [33]. Recently others have directly tested competing quantitative (local magnitude) and qualitative (spatial dissimilarity) models of sex modulation in autism, finding support for spatial dissimilarity models in cortical curvature but not thickness [20]. Further support of quantitative and qualitative models of sex neural modulation in autism include limited evidence of greater alterations in cortical thickness in autistic females [6] and sex specific spatial shifts of structural neural networks in autism [19].

Differences associated with autism in both cortical thickness and the trajectory of cortical thinning were distributed across all seven functional neural networks in both autistic males and females. Others have proposed that atypical functional organization and interconnections of the ventral attention (i.e., salience), frontoparietal (i.e., central executive), and default mode networks underpin a range of neuropsychiatric conditions including autism [60, 61]. This, ‘triple network model’, provides a transdiagnostic theory for neural processing underlying a range of behavioral and cognitive challenges. While we note effects of autism in both males and females within these three networks, sizeable portions of additional networks including the dorsal attention, limbic, somatomotor, and visual networks were also indicated, supporting recent findings that suggest autism does not universally arise from selectively targeted functional networks [62]. Also of note is that several of these findings were highly age dependent. This was most apparent in autistic females, who showed larger differences from both nonautistic females and autistic males in cortical thickness around 3 years of age compared to 11.5 years of age. This is consistent with findings that autistic individuals both experience regional differences in the timing of cortical thickening and thinning and that autistic differences are greater at younger ages [6, 28]. Collectively these results indicate autism is associated with cortical differences across multiple neural networks, and that many of these differences are highly dependent on both sex and developmental timing. A clear implication is that the age at which cortical differences in autism are evaluated can greatly influence findings. It also raises the interesting question of the mechanisms contributing to autistic females having greater differences in early compared to late childhood. Is this due to an altered developmental trajectory, a compensatory or some other process?

We also hypothesized that regions in which autism effects differed between the sexes would overlap with regions that are different between the sexes in neurotypical development. We did find that cortical regions that showed sex differences in autism effects overlapped to a significant degree with regions that showed sex differences in neurotypical development. This raises the possibility that alterations in the neural programs regulating neurotypical sex differentiation of the cerebral cortex contribute to sex differences in autism. This is notable given that biological sex has been implicated in developmental processes underpinning cortical thinning which include intracortical myelination, maturation and remodeling of dendritic trees, axonal innervation and collateralization, and vascularization [23]. Evidence for sex differentiation in these processes include increased expression of genes marking oligodendrocytes and somatosensory cortical pyramidal cells having been associated with increased thinning in males, but not females [63] and sex differences in dendritic arborization contributing to sex differences in subcortical brain volumes [64, 65]. Accordingly, the correspondence between cortical regions that show different effects of autism between males and females with regions that are different across the sexes in neurotypical development supports the theory that typical sex differential processes (e.g., sex steroid hormone exposure and regulation, sex-differential neural function) in the brain are linked to sex specific autism effects on cortical development [33]. Further support for this hypothesis has come from others who have noted shifts in functional connectivity in autism towards and away from patterns associated with their biological sex that overlap with gene expression patterns marking cell types involved in sex differential processes [66, 67].

Our final hypothesis was that there would also be areas of autistic cortical developmental differences shared across both sexes. This was based on the notion that the diagnostic features of autism are similar across the sexes and are likely mediated by the same brain regions. Somewhat surprisingly, in contrast to the widespread sex differences observed in early childhood and in the trajectory of cortical development, differences that were shared across sexes were limited to much smaller focal areas within select cortical regions. Thus, these findings support that differences in cortical thickness associated with autism between the sexes are larger than the commonalities between them.

It is still important to highlight, however, that while regions with shared autistic differences between males and females were limited to small focal areas, these fell within cortical regions that have often been highlighted in autism symptomology. These included regions of the parietal cortex, posterior cingulate cortex, and superior temporal gyrus, which are important for joint attention processing, disruption of which is associated with autistic development [68]. Both autistic males and females had thinner parahippocampal gyri across childhood. This region is important for visuospatial processing and was found to be smaller in autistic individuals across multiple studies in a recent metanalysis [69, 70]. We also observed that the right fusiform gyrus was thicker across sexes in autism across childhood. The fusiform gyrus has been widely implicated in autism for its role in facial perception and found to coactivate with the temporal pole during social perception tasks [71, 72]. The lateral occipital cortex, a region associated with alterations in autistic eye gaze and social communication [73, 74], was found to be thicker in autism across the sexes at ~3–5 years of age. In early childhood, for both autistic males and females, the superior frontal gyrus which is an important node in the salience network that shows altered functional connectivity in autism, was found to be thinner [75, 76]. Additionally, in later childhood we found autistic children of both sexes to have thinner cortex in the precentral gyrus, which has been reported to have altered functional connectivity with the posterior cingulate cortex linked to social impairments in autistic individuals [77].

The current study gains power from a longitudinal design enrolling near the age of diagnosis in early childhood and inclusion of one of the largest samples of autistic females. However, there are several limitations that must be taken into consideration in evaluating the results. First, the sample of autistic females at our latest reported timepoint (*n* = 17) remains relatively small. Larger samples and replication are needed to confirm associations between the development of the cortex with both autism and sex. Second, the age range of our sample does not allow us to investigate differences in rates of cortical development prior to 2 years of age, which is when cortical thickness is estimated to peak. Third, while we have previously reported on autistic sex differences in measures of cerebral volumes [14, 31, 32], due to the scope of the current work we focused solely on cortical thickness. Analyses of additional cortical measures such as surface area, gyrification, and contrast [17, 78] may provide further insights into sex specific autistic neurobiology. Moreover, we have portrayed average differences in cortical thickness and trajectory of cortical thinning in our autistic sample. However, a premise of our work is that there is substantial heterogeneity in the behavioral and biological underpinnings of autism in different individuals. Larger samples of autistic participants and methods specifically designed to capture individual variability [24] will be needed to determine if there are distinct subtypes or regional specialization of cortical development alteration that might associate with distinct behavioral profiles in autism. In this study, autistic males and females *did not* differ in average autism symptom severity or cognitive ability, and inclusion of developmental quotient at time of enrollment in models had negligible effects on results. Future studies should seek to study the association between cortical structure, behavior, cognition and/or co-occurring conditions. Additional longitudinal studies will be important as significant numbers of autistic individuals experience clinically meaningful change in autism symptoms and cognitive ability over time that may well be associated with differences in neural development [79–82].

In conclusion, we report significant sex differences in cortical development during childhood that are associated with autism in a longitudinal cohort that includes one of the largest MRI samples of autistic females to date. Sex differences in autistic cortical development were substantial and involved multiple neural networks. Interestingly, autistic females exhibited the greatest differences in cortical thickness from nonautistic females early in development around 3 years of age, but these differences attenuated across development. Larger differences associated with autism in females compared to males in regions that undergo neurotypical sex differentiation suggests that females have a larger liability threshold to develop autism, which may contribute to higher rates of autism in males. Effects of autism that were shared between the sexes were located in focal areas centered within select cortical regions that have often been implicated in the condition. Collectively, these results show that autistic males and females have substantial differences in autism neurophenotypes. This raises the possibility that alterations of the neural programs of cortical development that lead to neurotypical sex differentiation may be an important factor in the etiology of autism.

## Supplementary information

Supplementary Methods and Figures

Supplementary Tables

## Data Availability

The data underlying this article will be shared on reasonable request to the corresponding author.

## Code availability

All code used is freely available through public sources cited above and can be made available upon reasonable request to the corresponding author.
