# Supplementary material for: Sex differences in trajectories of cortical development in autistic children from 2–13 years of age
Source: Mol Psychiatry. 2024 May 16;29(11):3440–51. doi: 10.1038/s41380-024-02592-8 (PMC11541213; doi:10.1038/s41380-024-02592-8)

**Supplementary Methods**

*Participants*

All participants were required to be native English speakers, ambulatory, have no contraindications for MRI, no suspected vision or hearing problems, and no known genetic disorders or neurological conditions. An ASD diagnosis was confirmed at study entry by trained clinical psychologists using the Autism Diagnostic Observation Schedule-Generic (ADOS-G) (1) or ADOS-2 (2), the Autism Diagnostic Interview-Revised (ADI-R) (3) and DSM-IV-TR criteria (4). All autistic participants met ADOS-2 cutoff scores for either autism or ASD. In addition, they exceeded ADI-R cutoff scores for autism on either the social or communication subscale and were within two points of this criterion on the other subscale. ADOS calibrated severity scores were calculated to allow comparison of autism severity across participants tested with different ADOS modules (5). In 12 cases ADOS administration was not possible due to face masking requirements, in these cases ASD diagnosis was confirmed using the Brief Observation of Symptoms of Autism (BOSA) (6). At Time 1, TD individuals were screened for autism traits using the Social Communication Questionnaire (SCQ) (i.e., scores below 11) (7) and were required to have no first-degree relatives with an ASD diagnosis. The Mullen Scales of Early Learning (MSEL) (8) was used to assess developmental quotient (DQ) during participants first visit (Time 1). One autistic girl did not complete DQ assessment at Time 1, in this case the Differential Ability Scales 2^nd^ Edition (DAS-II) Early Years IQ assessment administered at Time 3 was used (9). TD children were excluded if they did not fall within two standard deviations on the MSEL. Informed consent was obtained from the parent or guardian of each participant. All aspects of the study protocol were approved by the University of California Davis Institutional Review Board.

*MRI Acquisition*

All MRI scanning was performed at the Imaging Research Center, UC Davis, Sacramento using a 3 Tesla Siemens Magnetom Trio MR system (Erlangen, Germany) with an 8-channel head coil. MRI scanning for Time 1-3 scans was performed during natural nocturnal sleep without sedation (10). High resolution T1 images were acquired using an MPRAGE sequence (1mm^3^ resolution, TR=2170ms, TE=4.86ms, 256x256x192mm FOV, 8:46 acquisition time). Time 4 scans were acquired when participants were awake utilizing principles of applied behavior analysis to improve compliance (11), in addition to a modified sequence to shorten scanning duration (1mm^3^ resolution, TR=2170ms, TE=3.5ms, FOV 256mm, FA 7, 5:10 acquisition time). Image distortion associated with the scanner was controlled for by scanning a calibration phantom (ADNI MAGPHAM, The Phantom Laboratory) at the end of each MRI session and subsequently applying distortion correction to each MPRAGE image (Image Owl, Inc, Greenwich, NY; <http://www.imageowl.com/>).

*Freesurfer Processing and Quality Control*

Individual estimates of cortical thickness were calculated based on cortical surface reconstructions of structural MRI scans using *Freesurfer* v7.1.1 (12,13). These methods have been extensively described elsewhere (14,15), and results have been validated histologically (16). All surface reconstructions were visually inspected for quality and, when appropriate, manual edits were performed to improve reconstruction quality.

The quality control process for cross-sectional MRI images involved a systematic evaluation of motion artifacts using a four-point qualitative scale: none, mild, moderate, and severe conducted by a single rater (D.C.). Supplementary Figure 2. This assessment identifies and categorizes the extent of motion-related distortions in the MRI scans including ghosting, blurring, extreme loss of signal and/or undesired strong signals (17). During the evaluation, images with no discernible motion artifacts are rated as "none," indicating high data quality. Those with minimal disturbances fall under the "mild" category, suggesting minor evidence of motion distortion, typically confined to one, small region of the brain that may not significantly impact surface reconstruction analysis. "Moderate" reflects a noticeable level of motion artifacts in several regions of the brain and though these cases may generate satisfactory surface reconstruction, they typically required extensive manual intervention and therefore were excluded to maintain the integrity of the data. "Severe" denotes substantial distortions that completely compromise the accuracy of subsequent image processing and analysis and were excluded from further processing.

Importantly, once scan’s level of quality was established, manual edits were employed to correct any inaccuracies found in the resulting white and pial surfaces. Inaccuracies include voxels that were inappropriately included or excluded from surfaces thereby diverting from anatomical accuracy. These were systematically corrected by viewing the problematic timepoint in the coronal plane. Each cross-section is then carefully examined, one hemisphere at a time, for inaccuracies and corrected by either adding/removing voxels or adding control points per *Freesurfer* website guidelines. Once both hemispheres have been examined and edited, the timepoint is rerun through the *Freesurfer* reconstruction pipeline so that the edits can be taken into consideration and more accurate surfaces can be generated.

At the time of analysis, 1115 MRI timepoints from eligible APP/GAIN participants had completed *Freesurfer* processing and quality control procedures. A total of 197 timepoints (15%) were excluded due to quality issues that could not be corrected by manual edits. Removing these timepoints resulted in the exclusion of 18 autistic males, 9 autistic females, 3 non-autistic typically developing (TD) male, and 3 TD females who are not described in the current study (48 total timepoints). At least one additional timepoint of sufficient quality was available for participants of the remaining 149 excluded timepoints, meaning these excluded timepoints did not result in a participant being excluded from the study as they had at least one additional quality timepoint available. Motion artifacts accounted for 92% of excluded scans, and the remaining 8% had other image quality issues (e.g., *b*0, hyperintensity artifacts) and/or failed to complete all automated *Freesurfer* preprocessing steps. There were no significant differences in the ratio of excluded timepoints for autistic males, autistic females, non-autistic males, and non-autistic females (χ^2^=0.29, *p*=0.96). While a larger proportion of Time 4 scans (and to a lesser degree Time 3 scans) were excluded (χ^2^=41.95, *p*<0.001) than from the earlier time points, there was no significant difference in the ratio between Time 4 autistic (38/101) and non-autistic scans (16/67) excluded (χ^2^=2.88, *p*=0.08).

Additionally, within our sample there was a higher proportion of autistic to non-autistic participants at Time 1 (2.01:1) compared to Time 4 (1.18:1) (*X*^2^=5.27, *p*=0.02). However, the ratio of autistic males to females did not significantly differ from Time 1 (2.31:1) to Time 4 (2.70:1) *X^2^*=0.11, *p*=0.73). Comparisons of between autistic participants who completed assessment and had quality MRI data for both Times 1 and 4 and those who did not have data at Time 4 showed no significant differences in ADOS CSS (*t*=-0.01, *p*=0.99) or DQ (*t*=-1.48, *p*=0.14). Additionally, there were no significant differences between autistic males who completed Time 4 v those who didn’t, nor autistic females who completed Time 4 v those who didn’t for either ADOS CSS or DQ (all *p*>0.15).

In addition to the above quality control procedures we report a quantitative measure of pre-edited reconstruction quality, i.e., the number of topological defects (i.e., *SurfaceHoles*) count returned by *Freesurfer* (14). Supplementary Table 46. Models including and excluding individual’s topological defect count as a nuisance covariate returned nearly identical statistical estimates. Supplementary Figure 3. Thus, we are confident that differences in *Freesurfer* reconstruction quality do not explain the current findings.

References

1. Lord C, Risi S, Lambrecht L, Cook EH, Leventhal BL, DiLavore PC, et al. The Autism Diagnostic Observation Schedule—Generic: A Standard Measure of Social and Communication Deficits Associated with the Spectrum of Autism. J Autism Dev Disord. 2000 Jun 1;30(3):205–23.

2. Lord C, Rutter M, DiLavore P, Risi S, Gotham K, Bishop S. Autism diagnostic observation schedule–2nd edition (ADOS-2). Los Angel CA West Psychol Corp. 2012;

3. Lord C, Rutter M, Le Couteur A. Autism Diagnostic Interview-Revised: a revised version of a diagnostic interview for caregivers of individuals with possible pervasive developmental disorders. J Autism Dev Disord. 1994;24(5):659–85.

4. Association AP. Diagnostic and Statistical Manual of Mental Disorders (DSM-5®). American Psychiatric Pub; 2013. 1520 p.

5. Gotham K, Pickles A, Lord C. Standardizing ADOS Scores for a Measure of Severity in Autism Spectrum Disorders. J Autism Dev Disord. 2009 May 1;39(5):693–705.

6. Dow D, Holbrook A, Toolan C, McDonald N, Sterrett K, Rosen N, et al. The Brief Observation of Symptoms of Autism (BOSA): Development of a New Adapted Assessment Measure for Remote Telehealth Administration Through COVID-19 and Beyond. J Autism Dev Disord. 2022;52(12):5383–94.

7. Rutter M, Bailey A, Lord C. The social communication questionnaire: Manual. Western Psychological Services; 2003.

8. Mullen EM. Mullen scales of early learning. AGS Circle Pines, MN; 1995.

9. Elliott C. Differential Ability Scales (2nd ed.). San Antonio, TX: Harcourt Assessment; 2007.

10. Nordahl CW, Simon TJ, Zierhut C, Solomon M, Rogers SJ, Amaral DG. Brief Report: Methods for Acquiring Structural MRI Data in Very Young Children with Autism Without the Use of Sedation. J Autism Dev Disord. 2008 Sep 1;38(8):1581–90.

11. Nordahl CW, Mello M, Shen AM, Shen MD, Vismara LA, Li D, et al. Methods for acquiring MRI data in children with autism spectrum disorder and intellectual impairment without the use of sedation. J Neurodev Disord. 2016 May 5;8(1):20.

12. Fischl B. FreeSurfer. NeuroImage. 2012 Aug 15;62(2):774–81.

13. Fischl B, Dale A. Measuring the thickness of the human cerebral cortex from magnetic resonance images. Proc Natl Acad Sci. 2000 12;97(20):11050–5.

14. Dale AM, Fischl B, Sereno MI. Cortical Surface-Based Analysis: I. Segmentation and Surface Reconstruction. NeuroImage. 1999 Feb 1;9(2):179–94.

15. Fischl B, Sereno MI, Dale AM. Cortical Surface-Based Analysis: II: Inflation, Flattening, and a Surface-Based Coordinate System. NeuroImage. 1999 Feb 1;9(2):195–207.

16. Rosas HD, Liu AK, Hersch S, Glessner M, Ferrante RJ, Salat DH, et al. Regional and progressive thinning of the cortical ribbon in Huntington’s disease. Neurology. 2002 Mar 12;58(5):695–701.

17. Zaitsev M, Maclaren J, Herbst M. Motion artifacts in MRI: A complex problem with many partial solutions. J Magn Reson Imaging. 2015;42(4):887–901.

**Supplementary Figure 1: Comparison of results including and excluding total brain volume and developmental quotient as covariates.**

**
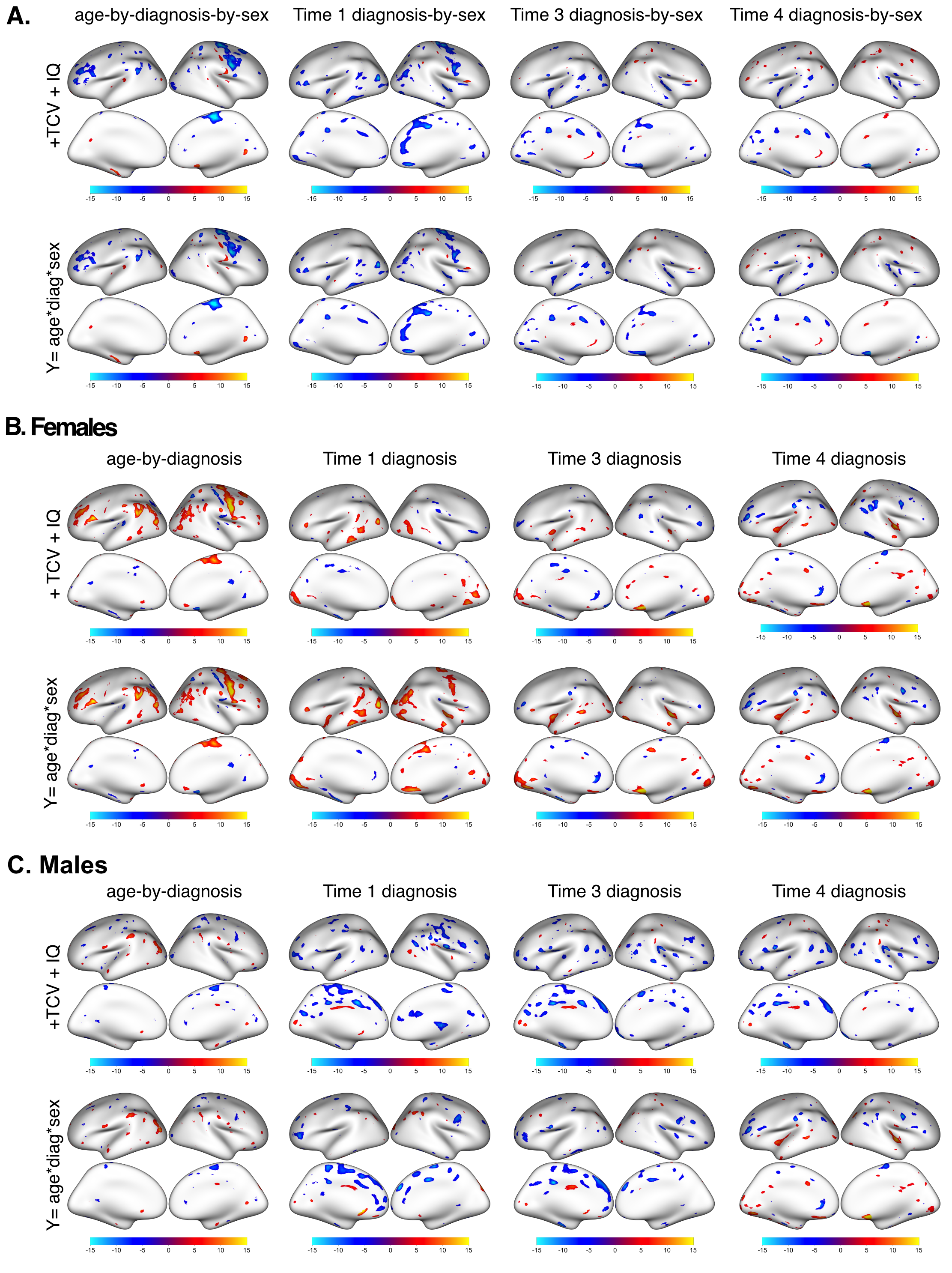
**

*Note*: Results of linear effects models of age-by-diagnosis-by-sex both including total cortical volume (TCV) and Time 1 developmental quotient (DQ) (first row of each A,B,C) and excluding TCV and DQ (second row of each A,B,C). All contrasts of interest were compared including A. age-by-diagnosis-by-sex and Times 1, 3, and 4 diagnosis-by-sex, as well as within sex age-by-diagnosis and Times 1, 3, and 4 diagnosis for both B. females and C. males. While some slight differences can be observed, inclusion of TCV and DQ had minimal effects on results.

**Supplementary Figure 2: Comparison of results including and excluding Freesurfer SurfaceHoles as a covariate.**


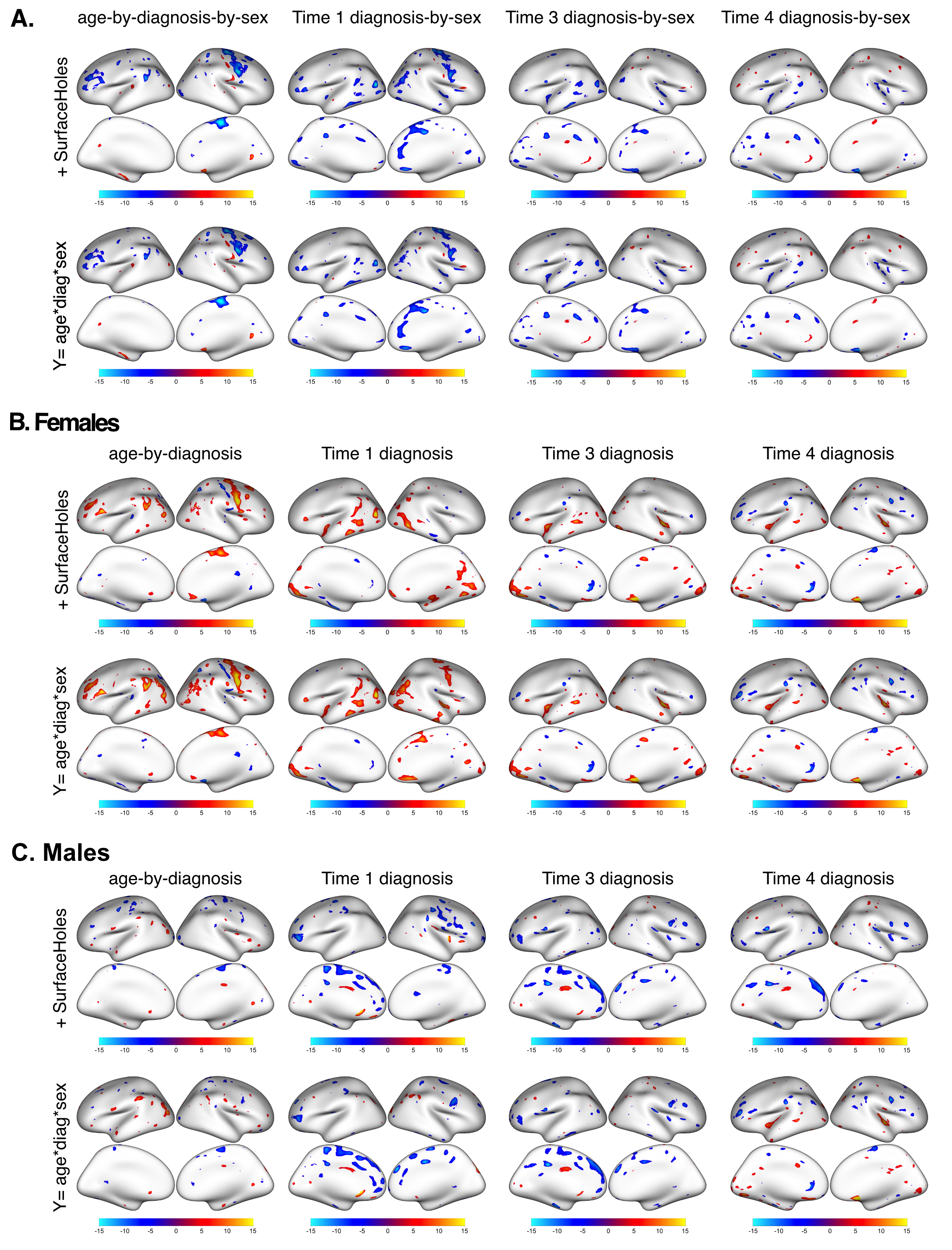


*Note*: Results of linear effects models of age-by-diagnosis-by-sex both including Freesurfer *SurfaceHoles* number of topological defects (first row of each A,B,C) and excluding *SurfaceHoles* number (second row of each A,B,C). All contrasts of interest were compared including A. age-by-diagnosis-by-sex and Times 1, 3, and 4 diagnosis-by-sex, as well as within sex age-by-diagnosis and Times 1, 3, and 4 diagnosis for both B. females and C. males. While some slight differences can be observed, inclusion of *SurfaceHoles* had minimal effects on results.

**Supplementary Figure 3: Motion artifact scale**


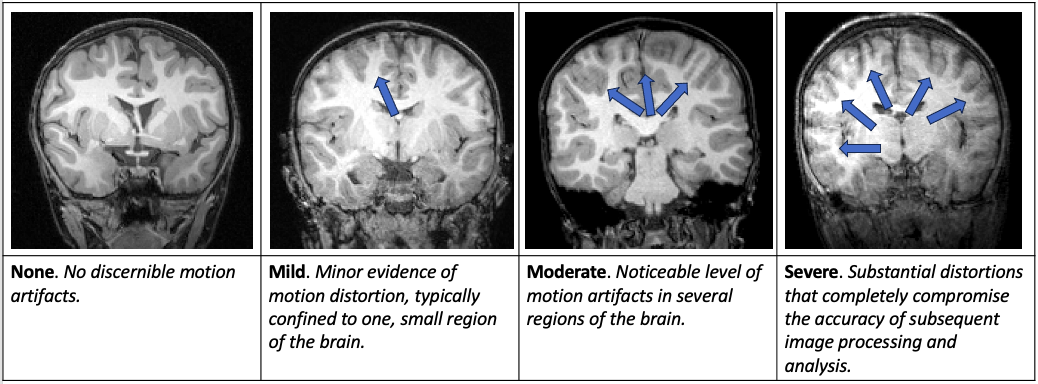

Supplement: Supplementary file 1 — Supplementary Methods and Figures [file 41380_2024_2592_MOESM1_ESM.docx]
